# Iohexol-based assessment of intestinal permeability in broilers challenged with *Eimeria maxima*, *Clostridium perfringens* or both

**DOI:** 10.3389/fphys.2024.1520346

**Published:** 2024-12-20

**Authors:** Ali Calik, Abhisek Niraula, Bingqi Dong, Candice E. C. Blue, Davis A. Fenster, Rami A. Dalloul

**Affiliations:** ^1^ Avian Immunobiology Laboratory, Department of Poultry Science, University of Georgia, Athens, GA, United States; ^2^ Department of Animal Nutrition and Nutritional Diseases, Faculty of Veterinary Medicine, Ankara University, Ankara, Türkiye

**Keywords:** iohexol, intestinal permeability, necrotic enteritis, broiler, tight junction

## Abstract

Impaired intestinal integrity in broilers reduces performance and health, highlighting the importance of accurately measuring intestinal permeability (IP) to maintain gut health. The objective of this study was to evaluate the efficiency of iohexol as an IP marker in broilers challenged with *Eimeria maxima*, *Clostridium perfringens*, or both during both peak challenge (day [d] 21) and recovery (d 28) periods. One-day-old male Ross 708 birds (n = 56) were distributed into 4 treatment groups: NC (no-challenge control); EM (challenged with 5,000 *E*. *maxima* sporulated oocysts/bird on d 15); CP (challenged with 1.0 × 10^8^ CFUs/bird of *C*. *perfringens* on d 19 and d 20); and EM + CP (challenged by co-infection of *E*. *maxima* and *C*. *perfringens* as described). On d 21 and d 28, each bird received an iohexol dose of 64.7 mg/kg body weight via oral gavage. One hour later, blood samples were collected from 14 birds (12 in EM) per group on d 21 and from 7 birds (6 in EM) on d 28. For lesion scoring and ileum collection, 7 birds per group (6 birds in EM) were sampled on each d 21 and d 28. Birds in the EM and EM + CP groups had lower body weight gain (BWG) compared to the NC and CP groups on d 19–21 (*P* ≤ 0.05). These birds also exhibited significantly greater lesion scores and markedly higher serum iohexol levels on d 21 (*P* ≤ 0.05). However, no significant differences in serum iohexol levels were observed among treatment groups following recovery on d 28. Moreover, significant differentials were observed in the mRNA abundance of key tight junction proteins (CLDN1, CLDN2, and ZO3), pro-inflammatory cytokines (IL-1β, IFNγ, and IL-22), and gut health markers (GLP2, OLFM4, and MUC2) in the EM and EM + CP groups compared to the NC and CP groups on d 21. In conclusion, this study demonstrates that iohexol is an effective marker for assessing IP in broilers under different enteric challenge conditions.

## 1 Introduction

The gastrointestinal tract (GIT) serves as the primary defense line and is the largest immune organ in poultry. An intact and functional intestinal barrier prevents the translocation of harmful substances, such as toxins, antigens, and pathogens, to the underlying tissue, while selectively allowing the absorption of nutrients, electrolytes, and water into the bloodstream (Schoultz and Keita, 2020). The mucin layer covering the surface of the mucosal membranes and tight junction (TJ) proteins, which seal the paracellular space between adjacent epithelial cells, are essential components of intestinal integrity (Emami et al., 2019). Disruption or dysfunction of intestinal integrity leads to increased intestinal permeability (IP), allowing the translocation of pathogens and undesired substances, which further triggers inflammation and a systemic inflammatory response (Vanuytsel et al., 2021). Pathogens, environmental stress, poor quality diet, mycotoxins, or microbial imbalance in the GIT could increase IP and compromise the barrier function in broilers. Considering the importance of gut health in broilers, especially as the industry is moving towards removing subtherapeutic antibiotics from the diets, increased attention to the concept of “leaky gut” led researchers to emphasize the need for reliable markers to assess IP.

Several markers including ^51^chromium-labeled ethylenediamine tetra-acetic acid (^51^Cr-EDTA), polyethylene glycols, ^14^Carbon (C)-mannitol, and ^14^C-inulin, lactulose-L-rhamnose, lucifer yellow, and horseradish peroxidase have been used for the assessment of IP (Schoultz and Keita, 2020). Even though these tests are considered as accurate, they are time consuming, lack standardization, and cannot be performed retrospectively (Camilleri, 2019; Seethaler et al., 2021). Additionally, the radioactivity of ^51^Cr-EDTA and the bacterial fermentation of sugar-based markers in the intestines have necessitated the investigation of new potential markers. Fluorescein isothiocyanate-dextran (FITC-d) is a fluorescently labeled sugar molecule with a molecular weight of 4 kDa. FITC-d is a well-documented paracellular probe and widely used in experiments conducted with broilers under various experimental challenge conditions in recent years (Hernandez-Patlan et al., 2019; Osho et al., 2023; Souza et al., 2024). However, a variety of factors influence the serum concentration of FITC-d including gavage dose, fasting period, and quantification protocol. Additionally, its light sensitivity can reduce the fluorescence signal and potentially affect measurement accuracy.

Iohexol is a water-soluble, non-ionic, non-radioactive radiological imaging contrast agent, which has long been recognized as an excellent marker for glomerular filtration rate in humans (Gaspari et al., 2018) and animals (Von Hendy-Willson and Pressler, 2011). Since it does not bind to serum proteins or metabolized, it is considered a safe, inert, and highly stable agent in biological samples (Gerova et al., 2011; Carrier et al., 2022). Due to its moderately high molecular weight (821 Da) and stability in the intestinal environment, it is also considered a potential IP marker for the assessment of intestinal integrity under various challenging conditions. Its potential use as an alternative to ^51^Cr-EDTA first time investigated by Andersen and Laerum (1995). Further studies, especially in humans with inflammatory bowel disease condition suggested that iohexol is a suitable marker for assessing intestinal integrity and barrier function (Gerova et al., 2011). Also, it has been successfully used for the assessment of IP in various animals including dogs (Frias et al., 2012), horses (McGilloway et al., 2023), and rats (Forsgård et al., 2016). In recent years, evaluation of intestinal barrier function using iohexol is also proven in clinically healthy birds (Wilhelm et al., 2020) and in birds challenged with coccidiosis (Rysman et al., 2023).

This study evaluated the efficiency of iohexol as an IP marker in broilers challenged with *Eimeria maxima*, *Clostridium perfringens*, or both during both peak challenge and recovery periods. Overall, the presented findings provide new insight into the usefulness of iohexol in monitoring intestinal permeability under various enteric challenge conditions.

## 2 Materials and methods

### 2.1 Birds, management, and challenge protocol

A total of 56 one-day-old male Ross 708 birds were individually weighed and wing tagged at initial placement and were raised on a standard starter diet for 15 days (d) with *ad libitum* access to feed and water. Subsequently, birds were distributed into 4 treatment groups each consisting of 14 birds. Three different challenge models along with a no-challenge control were used between d 15–28. The negative control (NC) served as a no-challenge control group. Birds in *E*. *maxima* (EM) group received an oral gavage of 5,000 *E*. *maxima* sporulated oocysts/bird on d 15. *C*. *perfringens* (CP) group were challenged with 1.0 × 10^8^ CFUs/bird of *C*. *perfringens* on d 19 and d 20. EM + CP model was challenged by co-infection of *E*. *maxima* and *C*. *perfringens* on similar days and at the same doses as described above. All birds were fed a standard corn-soybean meal grower diet during d 15–28. Birds were housed in clean battery cages for the duration of the trial (d 0–28). Ambient temperature was maintained at 33°C for the first 3 days, then gradually reduced approximately 3°C each week until it reached 22°C at the end of week 3, where it remained constant thereafter. The house was artificially ventilated and the lighting schedule was 24 h light for the first 3 days, reduced further to 23 h light and 1 h dark from d 4 to 7, and then kept constant at 20 h light and 4 h dark thereafter. All birds were weighed individually on d 0, 15, 19, 21, and 28. Feed intake (FI) was calculated by pen on d 15, 19, and 21. Body weight gain (BWG), FI, and feed conversion ratio (FCR) were subsequently calculated to evaluate growth performance.

### 2.2 Iohexol inoculation and sampling procedure

On d 21, individual bird BW was recorded and used to determine the exact dose of iohexol for each bird based on the rate of 64.7 mg/kg BW. Iohexol (Thermo Scientific Waltham, MA, United States) was diluted in sterile distilled water and given via oral gavage. The concentration of iohexol was determined based on previously published data to ensure accurate dosing for the assessment of IP (Rysman et al., 2023). This dosage was also used in broilers as a marker of renal function (Dhondt et al., 2019; Stroobant et al., 2020). After 1 h, blood samples (14 birds/group for NC, CP, and EM + CP and 12 birds/group for EM on d 21) were collected from the wing vein by direct venipuncture and stored in serum tubes. Samples were kept at room temperature for about 30 min and then centrifuged (1,500 × *g*, 15 min) to separate the serum samples which were stored at −80°C until analysis. Following blood collection, half of the birds in each group (7 birds/group for NC, CP, and EM + CP and 6 birds/group for EM) were euthanized by cervical dislocation for lesion scoring on d 21. Additionally, ileum samples were snap-frozen in liquid nitrogen to assess the mRNA abundance of TJ proteins, pro-inflammatory cytokines, and gut health markers. On d 28, the remaining birds (7 birds/group for NC, CP, and EM + CP and 6 birds/group for EM) were weighed individually and orally gavaged with iohexol to observe recovery. Blood collection, serum separation, tissue collection, and storage conditions were similar as described earlier.

### 2.3 Lesion scoring

Lesions in the jejunum and ileum were scored on d 21. Coccidiosis lesion scoring was performed according to the method of Johnson and Reid (1970) based on scores ranging from 0 (no gross lesions) to 4 (most severe lesions). NE lesion scoring was based on a 0–3 score, with 0 being normal and 3 being the most severe as previously described by Hofacre et al. (1998).

### 2.4 Sample preparation and serum iohexol determination

Serum samples were prepared by diluting 100 μL of serum with 100 μL of Milli-Q water. Each sample was then spiked with 25 μL of the internal standard iohexol-d5 (Cayman Chemicals, Ann Arbor, MI, United States) at a 100 μg/mL concentration. To precipitate proteins, 15 μL of 100% trifluoroacetic acid were added to each sample, followed by vortexing for 10 s. The samples were then centrifuged at 13,000 × *g* for 15 min. The resulting supernatant was carefully transferred to autosampler vials for subsequent analysis (Rysman et al., 2023).

Serum iohexol levels were analyzed by liquid chromatography-mass spectrometry (LC-MS) using an ACQUITY UPLC HSS T3 Column (2.1 mm × 50 mm, 1.8 µm, Waters Co., MA, United States). Mobile phase A consisted of 5% acetonitrile (ACN) and 95% water with 0.1% formic acid, and mobile phase B consisted of 95% ACN and 5% water with 0.1% formic acid. The flow rate was set at 0.4 mL/min and the injection volume was 5 µL. The gradient profile was set as follows: 0.0 min at 100% A, 0.5 min at 100% A, 6.0 min at 50% A, 6.1 min at 0% A, 8.0 min at 0% A, 8.1 min at 100% A, and 10.0 min at 100% A. Mass spectrometric detection was carried out on a Waters SYNAPT G2-Si High-Definition MS system with an electrospray ionization source operating in positive mode. The capillary voltage was set at 3.0 kV, the cone voltage at 25 V, and the source temperature at 120°C.

### 2.5 Total RNA extraction and reverse transcription

A 20–30 mg aliquot of ileum tissue was weighed into a 2-mL microcentrifuge tube and homogenized in 800 μL TRI Reagent (Zymo Research, Irvine, CA, United States) by a Bead Mill Homogenizer (VWR International, Radnor, PA, United States). Total RNA was extracted from the homogenate using the Direct-zol RNA Kits (Zymo Research) according to the manufacturer’s recommendations. Total RNA concentration was determined at optical density (OD) of 260 (NanoDrop One^C^ Spectrophotometer, Thermo Fisher Scientific, Waltham, MA, United States), and RNA purity was verified by evaluating the 260/280 OD ratios. RNA samples with a ratio in the range of 1.8–2.0 were considered high quality and were used to synthesize cDNA. RNA integrity was evaluated by gel electrophoresis on 1.5% agarose gel in 0.5 × TAE buffer. After extraction, 2 µg total RNA were used to synthesize first-strand cDNA using the qScript cDNA SuperMix synthesis kit (Quantabio, Beverly, MA, United States) according to the manufacturer’s recommendation and the cDNA was stored at −20°C.

### 2.6 Quantitative real-time PCR

The mRNA abundance of tight junction proteins [claudin (CLDN)1, CLDN2, CLDN3, zona occludens (ZO)1, ZO2, ZO3, occludin (OCLN), E-cadherin, junctional adhesion molecule (JAM)-A], pro-inflammatory cytokines [interleukin (IL)-1β, IL-22, and interferon gamma (IFNγ)], and intestinal health and function [glucagon-like peptide 2 (GLP2), olfactomedin 4 (OLFM4) and mucin-2 (MUC2)] related genes were determined by QuantStudio™ 3 Real-Time PCR System (Applied Biosystems, Waltham, MA, United States) using PowerTrack™ Fast SYBR Green Master Mix (Applied Biosystems). Details of primer sets are provided in Table 1. cDNA was diluted, and 2 μL of the diluted cDNA were added to each well of a 96-well plate. Subsequently, 8 μL of the real-time PCR master mix comprising 5 μL of PowerTrack™ Fast SYBR Green Master Mix, 0.5 μL of forward primer, 0.5 μL of reverse primer, and 2 μL of sterile nuclease-free water, were added to each well resulting in a final reaction volume of 10 μL. For the PCR reaction, samples were subjected to initial enzyme activation at 95°C for 2 min followed by 40 cycles of denaturation at 95°C for 5 s, annealing and extension at 60°C for 30 s. Product specificity was confirmed by analysis of the melting curves. The mRNA levels were analyzed using 18 S ribosomal subunit (18 S) gene as an endogenous control. Average mRNA abundance relative to 18 S for each sample was calculated using the 2^−ΔΔCt^ method (Livak and Schmittgen, 2001). The calibrator for each gene was the average ΔCt value from the NC group on a particular sampling day.

**TABLE 1:** Sequences of primer pairs used for amplification of target and reference genes.^a^

Gene^b^	Primer sequence	Size	Acc (reference)
*Tight Junction Proteins*			
CLDN1	F- GTGTTCAGAGGCATCAGGTATC R- GTCAGGTCAAACAGAGGTACAA	107	NM_001013611.2
CLDN2	F-CCTACATTGGTTCAAGCATCGTGA R- GATGTCGGGAGGCAGGTTGA	131	NM_001277622.1
CLDN3	F- CCCGTCCCGTTGTTGTTTTG R- CCCCTTCAACCTTCCCGAAA	126	NM_204202.1
ZO1	F- GGAGTACGAGCAGTCAACATAC R- GAGGCGCACGATCTTCATAA	101	XM_413773
ZO2	F- GCGTCCCATCCTGAGAAATAC R- CTTGTTCACTCCCTTCCTCTTC	89	NM_204918
ZO3	F- CAAAGCAAGCCGGACATTTAC R- GTCAAAATGCGTCCGGATGTA	78	XM_040692502.2
OCLN	F-CCGTAACCCCGAGTTGGAT R-ATTGAGGCGGTCGTTGATG	214	NM_205128.1
E-Cadherin	F-GAGTGGGAACCTGGATGGTG R- CATAATCCAGGCCCTTGGCTG	89	NM_001039258.3
JAM-A	F- TCACCTCGGAGACAAAGGAAGT R- ACGCAGAGCACGGGATGT	60	NM_001083366.1
*Cytokines*			
IL-1β	F-CGAGGAGCAGGGACTTTGC R-GAAGGTGACGGGCTCAAAAA	71	NM_204524.2
IL-22	F- GGGAAGAACAAAGCCATCGG R- CTCCCTTCTTTGGGGCATCA	82	NM_001199614.1
IFNγ	F- GCTCCCGATGAACGACTTGA R- TGTAAGATGCTGAAGAGTTCATTCG	63	NM_205149.2
*Intestinal Health and Function*			
GLP2	F- CGTGCCACAGCCATTCTTA R- AGCGGCTCTGCAAATGATTA	123	NM_001163248.1
OLFM4	F- ACCAGTGATGGGCACTTACG R- CCCTGACAAAGTTCGGTGGA	77	NM_001040463.2
MUC2	F- TTCATGATGCCTGCTCTTGTG R- CCTGAGCCTTGGTACATTCTTGT	93	XM_421035
*Housekeeping Gene*			
18 S	F-TCCCCTCCCGTTACTTGGAT R-GCGCTCGTCGGCATGTA	60	AF173612.1

^a^ Reference chicken gene sequences for forward (F) and reverse (R) (5′-3′) primers, the amplicon size (bp) and GenBank accession numbers used for the primer design are listed.

^b^ CLDN, claudin; ZO, zona occluden; OCLN, occludin; JAM-A, junctional adhesion molecule-A; IL, interleukin; IFNγ, interferon gamma; GLP2, glucagon-like peptide-2; OLFM4, olfactomedin 4; MUC2, mucin 2; 18 S, 18 S ribosomal RNA.

### 2.7 Statistical analysis

Data were subjected to a one-way analysis of variance (ANOVA) using the JMP Pro 17. When significant differences were noted, Tukey’s test was used to compare separated means. The influence of different challenge models on lesion scores were performed with the non-parametric Mann-Whitney U test. Statistical differences were considered significant at *p* ≤ 0.05. All figures were generated using GraphPad Prism 8 software (San Diego, CA, United States).

## 3 Results

### 3.1 Growth performance

Growth performance of broilers after the challenge period is shown in Table 2. Following *E*. *maxima* challenge until *C*. *perfringens* inoculation, no significant differences were observed among treatments in terms of BWG, FI, and FCR during d 15–19. Birds in EM and EM + CP treatments had lower BWG compared to NC and CP groups between d 19 and 21 (*p* < 0.001). Moreover, birds in EM and EM + CP groups exhibited statistically compromised FCR compared to NC and CP groups, with the EM + CP birds having the worst FCR, followed by EM during d 19–21 (*p* < 0.001). Birds in the CP group showed similar growth performance to NC birds during the challenge period.

**TABLE 2:** Broiler performance between d 0 and d 28.^a^

	Treatment groups^b^				Statistics	
Item	NC	EM	CP	EM + CP	SEM	*p*-value
BW						
d 0	47.35	46.93	47.21	47.00	0.25	0.934
d 15	562.4	569.7	561.4	573.0	6.45	0.908
d 0–19						
BW, g	854.9	839.7	839.4	854.7	10.22	0.912
BWG, g	807.5	792.9	792.2	807.7	10.18	0.913
FI, g	966.8	956.3	975.4	990.1	8.89	0.696
FCR	1.20	1.22	1.23	1.23	0.01	0.135
d 0–21						
BW, g	983.1^a^	906.5^b, c^	972.1^a, b^	893.4^c^	12.55	0.015
BWG, g	935.8^a^	859.9^b, c^	924.9^a, b^	846.4^c^	12.48	0.015
FI, g	1,154.6	1,086.7	1,173.9	1,115.5	15.40	0.158
FCR	1.23^b^	1.27^b^	1.27^b^	1.32^a^	0.01	0.016
d 15–19						
BWG, g	292.5	271.4	278.0	281.7	4.56	0.436
FI, g	359.9	335.6	356.0	356.6	5.02	0.352
FCR	1.23	1.28	1.28	1.27	0.01	0.223
d 19–21						
BWG, g	128.3^a^	55.7^b^	132.7^a^	38.7^b^	6.62	<0.001
FI, g	187.9^a^	130.4^b^	198.6^a^	125.5^b^	12.51	<0.001
FCR	1.47^c^	1.86^b^	1.50^c^	3.24^a^	0.27	<0.001
d 15–21						
BWG, g	420.8^a^	332.0^b^	410.7^a^	320.4^b^	8.64	<0.001
FI, g	547.7^a^	456.7^b^	554.6^a^	482.0^b^	16.58	0.014
FCR	1.30^b^	1.36^b^	1.35^b^	1.50^a^	0.03	0.010
d 21–28^c^						
BWG, g	669.1^a^	480.3^b, c^	580.0^a, b^	459.4^c^	24.49	0.003
d 0–28^c^						
BW, g	1,614.3^a^	1,375.3^b^	1,527.4^a, b^	1,348.3^b^	38.23	0.029
BWG, g	1,567.3^a^	1,328.5^b^	1,480.4^a, b^	1,301.1^b^	38.07	0.028

^a-c^Means with different superscripts in the same row are significantly different (*p* ≤ 0.05).

^a^ BW and BWG represent mean values of 14 birds per treatment for NC, CP, and EM + CP groups and 12 birds for the EM group during d 0–21. For d 21–28, the means were calculated from 7 birds for NC, CP, and EM + CP groups and 6 birds for the EM group; FI and FCR represent mean values of 2 replicate cages per treatment during d 0–21.

^b^ NC: negative control, no challenge; EM: challenged with 5,000 *E*. *maxima* sporulated oocysts/bird on d 15; CP: challenged with 1.0 × 10^8^ CFUs/bird of *C*. *perfringens* on d 19 and d 20; EM + CP: challenged with 5,000 *E*. *maxima* sporulated oocysts/bird on d 15 and 1.0 × 10^8^ CFUs/bird of *C*. *perfringens* on d 19 and d 20.

^c^ FI, and FCR, were not statistically evaluated since only one replicate cage left between d 21 and 28.

### 3.2 Lesion scores

**FIGURE 1:**
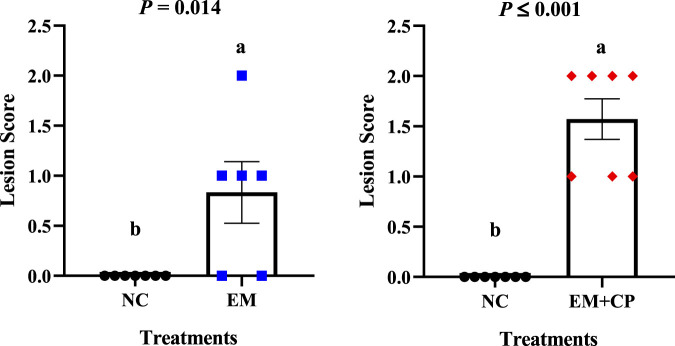
Lesion scores in the jejunum and ileum on d 21. Differences were determined by Mann-Whitney U test at 5% probability. NC: no-challenge control; EM: *E*. *maxima*; EM + CP: *E*. *maxima* + *C*. *perfringens*. ^(a,b)^Bars with different letters letters are significantly different (*p* ≤ 0.05).

*Eimeria maxima* and NE related lesion scores are shown in Figure 1. As expected, no lesions were observed in the NC group. Additionally, no visible lesions were seen in the CP group. Due to the different lesion scoring criteria used for the EM and EM + CP groups, their lesion scores were evaluated separately, revealing that both the EM (*p* = 0.014) and EM + CP (*p* ≤ 0.001) groups exhibited significantly greater lesion scores compared to the NC group on d 21.

### 3.3 Serum iohexol level

**FIGURE 2:**
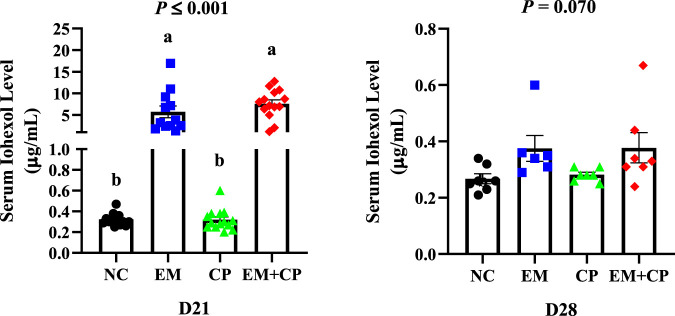
Serum iohexol levels of NC, EM, CP, and EM + CP treatments on d 21 and d 28. Data are presented as mean ± SEM with individual variables. NC: no-challenge control; EM: *E*. *maxima*; CP: *C*. *perfringens*; EM + CP: *E*. *maxima* + *C*. *perfringens*. Data are presented as mean ± SEM (n = 14 birds/group for NC, CP, and EM + CP while n = 12 birds/group for EM on d 21 and n = 7 birds/group for NC, CP, and EM + CP while n = 6 birds/group for EM on d 28). ^(a,b)^Bars with different letters are significantly different (*p* ≤ 0.05).

Serum iohexol level of broilers during the peak challenge (d 21) and recovery (d 28) periods are shown in Figure 2. On d 21, a significant difference in serum iohexol levels were observed among the treatment groups (*p* ≤ 0.001). The EM (5.73 μg/mL) and EM + CP (7.60 μg/mL) groups exhibited markedly greater serum iohexol levels compared to NC (0.32 μg/mL) and CP (0.32 μg/mL) groups. Although there were no significant differences among the treatment groups in terms of serum iohexol level on d 28, the EM (0.37 μg/mL) and EM + CP (0.37 μg/mL) groups tended to have higher (*p* = 0.070) levels compared to NC (0.27 μg/mL) and CP (0.28 μg/mL) groups.

### 3.4 mRNA abundance of gut integrity and immune response-related genes in the ileum on d 21

**FIGURE 3:**
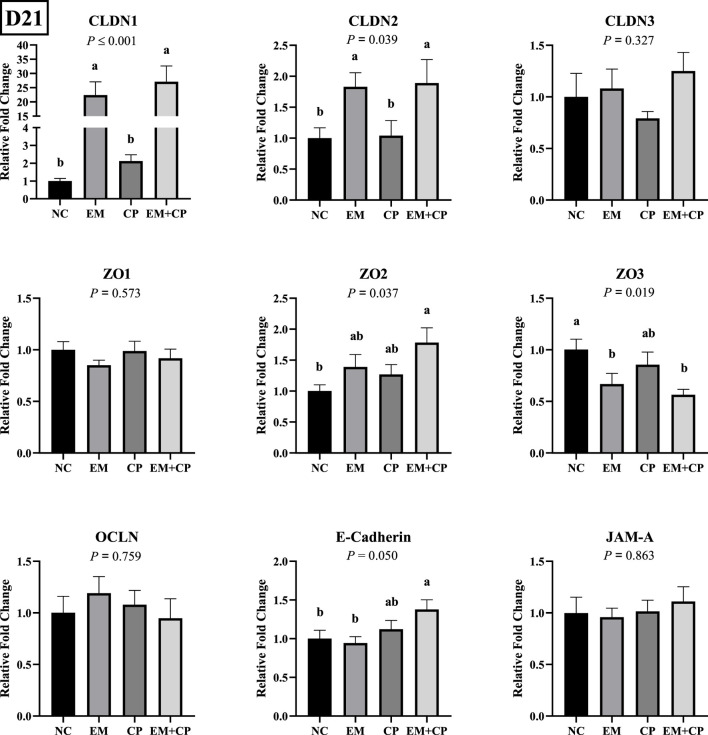
Relative mRNA abundance of TJ protein-related genes on d 21 in the ileum of birds challenged with *E*. *maxima*, *C*. *perfringens*, or both. The relative mRNA abundance of these targets was determined by qPCR and analyzed by 2^−ΔΔCt^ method using NC group as a calibrator. Data are presented as mean ± SEM (n = 7 birds/group for NC, CP, and EM + CP and n = 6 birds/group for EM). ^(a,b)^Bars with different letters are significantly different (*p* ≤ 0.05).

Effects of EM, CP, or EM + CP challenge on mRNA abundance of TJ protein-related genes in the ileum of birds are shown in Figure 3. CLDN1 (*p* ≤ 0.001) and CLDN2 (*p* = 0.039) mRNA abundance were significantly higher in EM and EM + CP groups compared to NC and CP groups. mRNA abundance of ZO2 showed a significant difference among treatment groups (*p* = 0.037) where the EM + CP treatment resulted in the highest fold change compared to NC group. mRNA abundance of ZO3 was significantly lower in EM and EM + CP groups compared to NC group but not significantly different from the CP group (*p* = 0.019). Moreover, E-cadherin mRNA abundance was significantly higher in EM + CP group compared to NC and EM groups but similar to CP group. No significant differences were observed in mRNA abundance of CLDN3, ZO1, OCLN, and JAM-A among the treatment groups on d 21.

**FIGURE 4:**
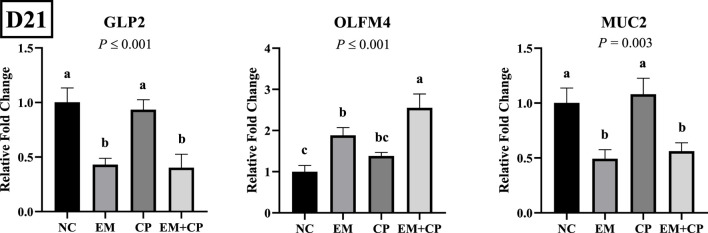
Relative mRNA abundance of GLP2, OLFM4, and MUC2 on d 21 in the ileum of birds challenged with *E*. *maxima*, *C*. *perfringens*, or both. The relative mRNA abundance of these targets was determined by qPCR and analyzed by 2^−ΔΔCt^ method using NC group as a calibrator. Data are presented as mean ± SEM (n = 7 birds/group for NC, CP, and EM + CP and n = 6 birds/group for EM). ^(a–c)^Bars with different letters are significantly different (*p* ≤ 0.05).

**FIGURE 5:**
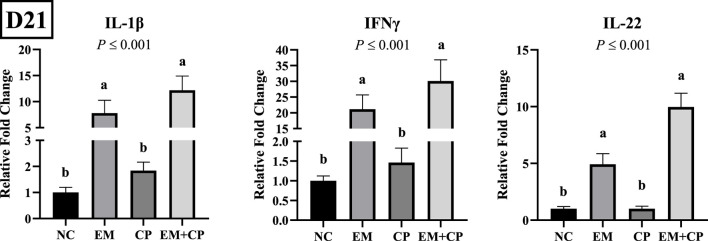
Relative mRNA abundance of IL-1β, IFNγ, and IL-22 on d 21 in the ileum of birds challenged with *E*. *maxima*, *C*. *perfringens*, or both. The relative mRNA abundance of these targets was determined by qPCR and analyzed by 2^−ΔΔCt^ method using NC group as a calibrator. Data are presented as mean ± SEM (n = 7 birds/group for NC, CP, and EM + CP and n = 6 birds/group for EM). ^(a,b)^Bars with different letters are significantly different (*p* ≤ 0.05).

The relative mRNA abundances of GLP2, OLFM4, and MUC2 in the ileum of the birds challenged with EM, CP, or EM + CP are shown in Figure 4. GLP2 (*p* ≤ 0.001) and MUC2 (*p* = 0.003) mRNA abundance were significantly lower in the EM and EM + CP groups compared to the NC and CP groups. OLFM4 mRNA abundance was significantly greater in EM and EM + CP groups compared to NC group (*p* ≤ 0.001). However, CP birds had similar levels of OLFM4 mRNA compared to NC and EM birds. As shown in Figure 5, mRNA abundances of IL-1β, IFNγ, and IL-22 were significantly higher in EM and EM + CP groups compared to NC and CP groups in the ileum on d 21 (*p* ≤ 0.001).

### 3.5 mRNA abundance of gut integrity and immune response-related genes in ileum on d 28

**FIGURE 6:**
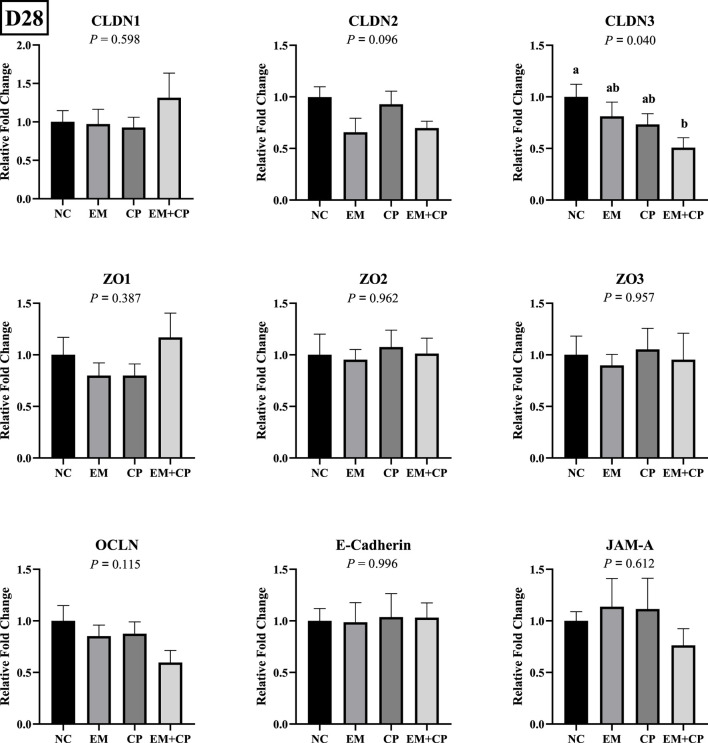
Relative mRNA abundance of TJ protein-related genes on d 28 in the ileum of birds challenged with *E*. *maxima*, *C*. *perfringens*, or both. The relative mRNA abundance of these targets was determined by qPCR and analyzed by 2^−ΔΔCt^ method using NC group as a calibrator. Data are presented as mean ± SEM (n = 7 birds/group for NC, CP, and EM + CP and n = 6 birds/group for EM). ^(a,b)^Bars with different letters are significantly different (*p* ≤ 0.05).

CLDN3 mRNA level was significantly lower in the EM + CP group compared to the NC group. However, EM and CP groups exhibited similar CLDN3 mRNA abundance compared to NC and EM + CP groups. CLDN1, CLDN2, ZO1, ZO2, ZO3, OCLN, E-cadherin, and JAM-A, did not show statistically significant differences in mRNA abundance among the treatment groups on d 28 (Figure 6).

**FIGURE 7:**
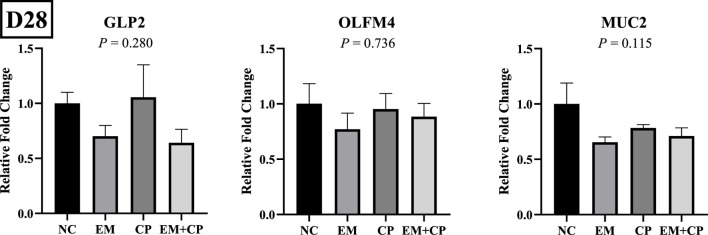
Relative mRNA abundance of GLP2, OLFM4, and MUC2 on d 28 in the ileum of birds challenged with *E*. *maxima*, *C*. *perfringens*, or both. The relative mRNA abundance of these targets was determined by qPCR and analyzed by 2^−ΔΔCt^ method using NC group as a calibrator. Data are presented as mean ± SEM (n = 7 birds/group for NC, CP, and EM + CP and n = 6 birds/group for EM).

**FIGURE 8:**
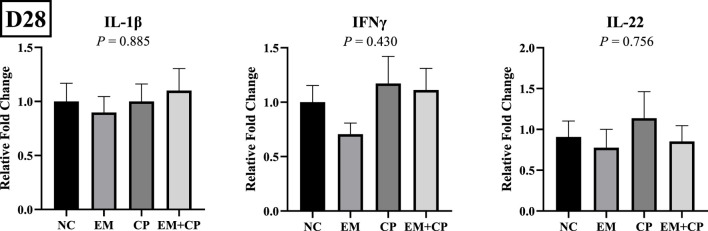
Relative mRNA abundance of IL-1β, IFNγ, and IL-22 on d 28 in the ileum of birds challenged with *E*. *maxima*, *C*. *perfringens*, or both. The relative mRNA abundance of these targets was determined by qPCR and analyzed by 2^−ΔΔCt^ method using NC group as a calibrator. Data are presented as mean ± SEM (n = 7 birds/group for NC, CP, and EM + CP and n = 6 birds/group for EM).

The mRNA abundance of genes associated with gut health, including GLP2, OLFM4, and MUC2, did not exhibit significant differences across the treatment groups on d 28 (Figure 7). Similarly, the mRNA abundance levels of IL-1β, IFNγ, and IL-22 showed no statistically significant differences among the treatment groups on d 28 (Figure 8).

## 4 Discussion

Impaired intestinal integrity caused by various factors in broilers is directly associated with reduced growth performance. Especially after the ban on the use of subtherapeutic antibiotics in diets, maintaining optimal intestinal health has become a fundamental goal in the industry for preventing diseases, supporting immune function, and ensuring optimum digestion and nutrient absorption. Accurate measurement of IP with reliable markers is one of the key strategies for assessing gut health and could help to implement effective interventions to enhance overall performance and health. Therefore, the current study investigated the use of iohexol as an IP marker in broilers challenged with *E*. *maxima*, *C*. *perfringens*, or both during both peak challenge and recovery periods.

Coccidiosis and NE challenges compromise the integrity of intestinal epithelial lining, leading to triggered inflammatory response and increased IP, which further results in impaired growth performance (Calik et al., 2019; Emami et al., 2019; Hansen et al., 2021; Blue et al., 2023; Froebel et al., 2024). In the current study, we successfully established subclinical *E*. *maxima* and NE challenge models to examine the TJ disruption and assess the efficacy of iohexol as an external IP marker during both peak challenge and recovery periods. According to our findings, birds challenged with *E*. *maxima* alone or with *E*. *maxima* and *C*. *perfringens* together showed reduced growth performance and exhibited significantly greater intestinal lesions compared to other treatments on d 21. Besides, birds in the CP group showed similar growth performance with NC birds during the challenge period. This was somewhat an expected outcome since *C*. *perfringens* needs several predisposing factors such as coccidiosis, intestinal dysbiosis, or poor-quality/poorly balanced diets to induce NE (Emami and Dalloul, 2021). In line with the performance and lesion score results, birds in EM and EM + CP groups exhibited significantly higher serum iohexol levels compared to NC and CP groups on d 21. Consistent with our observations, a recent study demonstrated that birds challenged with different strains of *Eimeria* spp. exhibited significantly greater serum iohexol levels on d 20 and d 21 (Rysman et al., 2023). Our findings align with previous studies in humans, horses, dogs, and rats, where iohexol effectively reflected changes in IP, supporting its role in assessing intestinal integrity under various conditions (Klenner et al., 2009; Gerova et al., 2011; Frias et al., 2012; Forsgård et al., 2016; McGilloway et al., 2023). Moreover, the current study also is the first to measure serum iohexol levels after the recovery phase (d 28) to improve our understanding and efficacy of iohexol as an IP marker. Although the EM and EM + CP groups showed slightly higher serum iohexol levels compared to the NC and CP groups, these results indicate a possible recovery of intestinal integrity over time in the challenged groups.

Epithelial cells regulate selective permeability through two main routes: the transcellular and paracellular pathways. The transcellular pathway, mediated by transporters and channels, manages nutrient absorption, while the paracellular pathway allows transport between cells, controlled by tight and adherence junctions (Suzuki, 2013). Disruption of TJ proteins result in a loss of barrier function, increasing permeability via the paracellular pathway (Berkes et al., 2003; Horowitz et al., 2023). When intestinal barrier integrity is compromised, the permeability of TJs increases, allowing external markers to pass through the paracellular pathway, where they enter the bloodstream and can be quantified in serum as a measure of IP (Schoultz and Keita, 2020). The paracellular route used by ions, water and larger hydrophilic compounds from ∼400–600 Da up to 10–20 kDa and that cannot cross transcellularly (Vanuytsel et al., 2021). Most studies on paracellular transport identify two distinct pathways regulated by TJs: (1) the pore pathway, which is a high-capacity, charge-selective route that permits the passage of small ions and uncharged molecules, and (2) the leak pathway, a low-capacity route that allows larger ions and macromolecules to pass, regardless of their charge (Vanuytsel et al., 2021; Horowitz et al., 2023). The pore pathway is primarily controlled by CLDN proteins, which form selective pores that regulate ion permeability. In contrast, the leak pathway is governed by OCLN and ZO proteins, which manage the transport of larger molecules, including proteins and peptides (Watson, 2015).

In the present study, significant alterations were observed in the mRNA abundance of key TJ proteins (CLDN1, CLDN2, and ZO3), pro-inflammatory cytokines (IL-1β, IFNγ, and IL-22), and gut health markers (GLP2, OLFM4, and MUC2) in the EM and EM + CP treatment groups compared to the NC and CP groups on d 21. CLDN1 plays a crucial role in maintaining selective permeability by regulating the passage of ions, water, and small molecules while preventing the leakage of harmful substances such as toxins and pathogens from the gut lumen into the underlying tissues (Lu et al., 2013). Interestingly, the current study revealed significantly higher CLDN1 mRNA abundance in the EM and EM + CP groups compared to the NC and CP groups, despite its role as a pore-sealing TJ protein. Due to its physiological role as a pore-sealing TJ-protein, the increase observed in the EM and EM + CP treatments is somewhat unexpected. However, greater CLDN1 mRNA abundance in the small intestine of broilers under *Eimeria* spp. and NE challenge conditions has been documented (Bortoluzzi et al., 2019; Gharib-Naseri et al., 2021; Goo et al., 2023). The elevation of pro-inflammatory cytokines observed in this study may contribute to the induction of CLDN1 mRNA expression, aligning with the findings of Pope et al. (2014). Additionally, this increase may be associated with the anti-apoptotic effects of CLDN1, providing protective benefits in challenged birds (Akasaka et al., 2010; Gharib-Naseri et al., 2021). Therefore, increased CLDN1 expression does not necessarily indicate enhanced TJ function, as its regulation may reflect a compensatory response to epithelial damage rather than a direct improvement in barrier integrity. Unlike CLDN1, CLDN2 is a pore-forming TJ protein and increased levels would directly lead to hyperpermeability and worsen barrier function (Yoseph et al., 2016; Oami et al., 2024). *In vivo* and *in vitro* studies have implicated IL-1β, IL-6, IL-13, IL-22, and TNFα as potential enhancers of CLDN2 (Heller et al., 2005; Mankertz et al., 2009; Suzuki et al., 2011; Tsai et al., 2017; Raju et al., 2020). Additionally, ZO3, a critical scaffold protein that links transmembrane TJ proteins (such as claudins) to the actin cytoskeleton, showed reduced mRNA abundance in response to EM and EM + CP challenges. At the molecular level, the first pore is mainly regulated by CLDNs and the latter by the OCLN and the ZO family (Vanuytsel et al., 2021). We did not observe a noticeable difference in OCLN and ZO1 in mRNA abundance during the peak infection. This lack of change is believed to be related to the subclinical challenge model used in the current study, which is consistent with previous findings (Emami et al., 2019; Emami et al., 2020; Goo et al., 2023). Observed changes in TJ proteins and pro-inflammatory cytokines were accompanied by a decrease in GLP2 and MUC2 mRNA abundance, along with an increase in OLFM4 abundance in both the EM and EM + CP groups compared to NC group. GLP2 promotes growth and function of the intestinal epithelium and improves the intestinal barrier function (Abdalqadir and Adeli, 2022). A previous study reported a significant negative association between plasma GLP2 and intestinal permeability (Cani et al., 2009). Decreased MUC2 levels are often attributed to goblet cell damage induced by intestinal challenges (Gharib-Naseri et al., 2021). Moreover, elevated CLDN1 mRNA level has been suggested to activate Notch signaling, which inhibits goblet cell differentiation and further contributes to reduced mucin production (Pope et al., 2014). OLFM4 is a stem cell marker and plays a crucial role in maintaining gut homeostasis and regenerating damaged tissue (Kinstler et al., 2024). Increased mRNA level of OLFM4 during peak infection, particularly in EM + CP treatment, is likely related to the promotion of intestinal epithelial cell regeneration in response to the increased intestinal inflammation (Xing et al., 2024). Therefore, all these observed changes in the ileum clearly show the disruptions of intestinal integrity and increase in IP during peak infection. However, during the recovery period, the mRNA levels of both TJ proteins (except CLDN3), pro-inflammatory cytokines, and intestinal health-related markers had largely returned to baseline levels, with no significant differences observed between the various treatment groups.

The impact of intestinal challenges such as *Eimeria* spp. or NE on intestinal TJ proteins, pro-inflammatory cytokines, and gut health markers has been extensively investigated in previous studies. However, there are few methodologies available for assessing IP with external markers in avian species. Although there is considerably more information available on FITC-d (Liu et al., 2021), studies on iohexol as an IP marker in broilers remain limited. It is known that a reliable IP marker should exhibit low absorption through the intact intestinal barrier and increased absorption through inflamed intestinal mucosa (Schoultz and Keita, 2020). In the current study, we observed a significant elevation in serum iohexol levels during the peak challenge period, which subsequently returned to near baseline levels during the recovery phase. Additionally, an IP marker should be small, non-metabolizable, water-soluble, has low protein binding, be chemically stable, and detectable by reliable methods. The radiographic contrast agent iohexol, with a molecular weight of 821 Da, is significantly smaller than FITC-d (4 kDa), a commonly used intestinal marker in broilers. The smaller molecular weight of the iohexol could be advantageous in evaluating the paracellular permeability in subclinical challenge models or during the early phase of the *Eimeria* spp. or NE challenges (Rysman et al., 2023). Moreover, detection methods for iohexol, such as HPLC or mass spectrometry, may enable more accurate and quantitative measurements. However, further comparative studies are needed to confirm these advantages and determine its broader applicability in broilers.

Overall, we observed an increase in serum iohexol levels during peak infection, along with significant changes in performance parameters, lesion scores, mRNA abundance of key TJ proteins, pro-inflammatory cytokines, and gut health markers in EM and EM + CP birds compared to NC and CP birds. During the recovery period, the lack of noticeable differences in these parameters likely reflects a recovery of intestinal integrity and barrier function. In line with our current findings, we conclude that iohexol can be effectively used as an IP marker to assess alterations in intestinal integrity in broilers.

## Data Availability

The original data presented in the study are included in this article, further inquiries can be directed to the corresponding author.
